# Biomarkers of delayed graft function as a form of acute kidney injury in kidney transplantation

**DOI:** 10.1038/srep11684

**Published:** 2015-07-15

**Authors:** Jolanta Malyszko, Ewelina Lukaszyk, Irena Glowinska, Magdalena Durlik

**Affiliations:** 1 2nd Department of Nephrology, Medical University, Bialystok, Poland; 2 Department of Nephrology and Transplantation Medicine, Warsaw Medical University, Poland

## Abstract

Renal transplantation ensures distinct advantages for patients with end-stage kidney disease. However, in some cases early complications can lead to allograft dysfunction and consequently graft loss. One of the most common early complications after kidney transplantation is delayed graft function (DGF). Unfortunately there is no effective treatment for DGF, however early diagnosis of DGF and therapeutic intervention (eg modification of immunosuppression) may improve outcome. Therefore, markers of acute kidney injury are required. Creatinine is a poor biomarker for kidney injury due principally to its inability to help diagnose early acute renal failure and complete inability to help differentiate among its various causes. Different urinary and serum proteins have been intensively investigated as possible biomarkers in this setting. There are promising candidate biomarkers with the ability to detect DGF. We focused on emerging biomarkers of DGF with NGAL is being the most studied followed by KIM-1, L-FABP, IL-18, and others. However, large randomized studies are needed to establish the value of new, promising biomarkers, in DGF diagnosis, prognosis and its cost-effectiveness.

During calendar year 2012, 17,305 kidney transplants, including kidney-alone and kidney plus at least one additional organ, were performed in the United States (http://www.usrds.org/2014/view/v2_06.aspx accessed March 29th, 2015). Of these kidney transplants, 5,617 were identified as coming from living donors and 11,535 from deceased donors. In 2014 in Poland 1111 kidney transplantations were performed, including 37 simultaneous pancreas-kidney transplantations (http://www.poltransplant.org.pl/, accessed March 29^th^, 2015). Of these, in Poland 1056 kidney transplant came from deceased donors and 55 came from living donors. Renal transplantation ensures distinct advantages for patients with end-stage kidney disease. However, in some cases early complications can lead to allograft dysfunction and consequently graft loss. One of the most common early complications after kidney transplantation is delayed graft function (DGF), defined as the necessity of dialysis during the first week after renal transplantation. It is a manifestation of acute kidney injury (AKI) distinctive for transplant process. The prevalence of DGF is significantly higher in extended donor criteria (EDC) kidneys and in donation after cardiac death (DCD)^1^,^2^. 4–10% of live donor and 5–50% of deceased donor kidney transplant is complicated by AKI leading to DGF. The risk factors of DGF are donor-derived (i.e. age, duration of ischemia) and recipient-derived (ischemia-reperfusion injury, immunological response, immunosuppressive medications)^3^. The incidence of DGF increases as a result of growing frequency of donation organs from non-heart-beating donors and extended criteria donor^4^. DGF is associated with increased risk of graft loss and death^5^. Unfortunately there is no effective treatment for DGF, however early diagnosis of DGF and therapeutic intervention (eg modification of immunosuppression) may improve outcome. Therefore markers of acute kidney injury are required.

The most frequent cause of DGF is acute tubular necrosis (ATN)^6^. The process underlying the cause of ATN is complex and includes several periods: prerenal (the impairment of perfusion), then onset, deepening and sustain of damage and finally repair of damage. There are some identified risk factors that increase the incidence of ATN such as cold ischemia time more than 24 hours, prior sensitization in retransplanted patients, the type of dialysis performed prior to transplantation and the quality of donor^7^,^8^,^9^,^10^,^11^,^12^,^13^,^14^.

The causes of acute tubular necrosis include: epithelial and endothelial damage due to tubules occlusion, impairment of microvascular flow, immunological and inflammation processes.

Main histological changes of ATN are: merging and loss of tubular epithelial cells, focal dilatation of proximal tubules, partial occlusion of tubular lumens by cellular debris and multiple mitoses. The impairment of renal function is usually more marked than histological abnormalities. Moreover, microvascular endothelium is involved in pathogenesis of ATN^15^,^16^. Due to serious possible consequences of ATN and the following DGF there is a necessity of identifying early biomarkers of AKI in this situation. This paper presents several promising biomarkers that may be potential indicators of DGF (Table 1).

## Creatinine

Historically, the first marker used to formally assess kidney function was urea, the major form of nitrogenous waste in the body. Richard Bright in 1827 was the first describe the association between urea accumulation in the blood and its decrease in the urine among individuals with diseased kidneys^17^. In the early 1900s blood urea nitrogen (BUN) quantification was eventually introduced into clinical medicine as a diagnostic test^18^. Serum creatinine supplanted BUN for the assessment of kidney function in the mid-1900s and remains the most widely used laboratory test to estimate GFR^19^. Today the “gold standard” in assessment of kidney function and estimation of glomerular filtration rate (GFR) is serum creatinine level, however, it is still imperfect due to the impact of a number of additional factors including diet, muscle mass and metabolism, gender, drugs, hydration status, where normal serum creatinine is of limited use^20^. In addition, normal serum creatinine level have a wide range of the reference values^21^. Moreover, the estimation of GFR in AKI is adulterated. It stems from the fact, that plasma creatinine concentration increase in case of failure more than 50% of kidney function. In addition, acute kidney injury, as defined by serum creatinine, may not reflect tubular injury. On the other hand, the absence of serum creatinine changes does not preclude the presence of tubular injury. Thus, a number of acute and chronic renal diseases can occur without an increase in serum creatinine^22^. Despite widespread acknowledgment of the limitations of serum creatinine, definitions of AKI continue to rely on this substance as a diagnostic standard, perhaps because of the historical absence of validated primary biomarkers of kidney injury^23^,^24^,^25^. Moreover, researchers still use changes in serum creatinine as a gold standard to test the usefulness the novel tubular biomarkers of kidney injury in clinical trials^26^. It is still suboptimal or less ideal approach, but so far there is no gold standard and creatinine may be considered as imperfect or “silver standard” in the assessment of kidney function.

### Novel biomarkers

Marker is an objectively measurable substance. Furthermore it is evaluated as an indicator of normal or pathological process. Besides, it is useful to assess the response to treatment. Biomarkers general utility refers to several functions: diagnosis, efficacy and safety. Biomarkers can be applied to early detection and risk stratification. Their most widespread use refers to monitoring effectiveness of therapies and prediction health status. An ideal biomarker does not still exist. It should be easy, safe and inexpensive to measure, relatively stable in the population and reflect the effectiveness of treatment.

As far as renal damage is concerned, biomarkers may be detected not only in urine but in serum as well. There are several potential mechanisms resulting in appearance of biomarkers in urine, for instance filtration across the glomerular basement membrane, passive or active release, resorption or catabolism.

First research on biomarkers focused on urinary tubular enzymes including proximal renal tubular epithelial antigen (HRTE-1), alpha-Glutathione S-transferase (alpha-GST), pi-Glutathione S-transferase (pi-GST), gamma-Glutamyltranspeptidase (gamma-GT), alanine aminopatidase (AAP), lactate dehydrogenase (LDH), N-acetyl-beta-glucosaminidase (NAG), alkaline phosphatase (ALP). Their advantage over creatinine results from an earlier release (from 12 hours to 4 days prior a detectable increase in serum creatinine)^25^. Their drawback is the inability to distinguish acute tubular necrosis from prerenal cause of acute kidney injury. It stems from the lack of validated cut-off points. There are also several low-molecular-weight urinary proteins, which are not normally secreted in kidney. Hence their emergence in urine indicates renal damage. This group of proteins includes: alpha1-microglobulin (alpha1-M), beta2-microglobulin (beta2-M), retinol binding protein (RBP), adenosine deaminase binding protein (ABP) and urinary cystatin C. The increase of these biomarkers excretion does not necessary has to be associated with irreversible and persistent renal damage. In certain cases, it may relate only to mild and reversible dysfunction. Their advantage is an indication of a possible pathomechanism likely to be a cause of acute kidney injury. Unfortunately, both tubular enzymes as well as low-molecular weight proteins do not possess enough specific and standardized assays^27^. In addition, during DGF when diuresis is suboptimal, urinary biomarkers are of no value. Therefore, we may employ serum biomarkers may be predictive of diagnosis of DGF or prognosis of outcome.

Novel tubular injury biomarkers may revolutionize the diagnosis of acute kidney injury, including DGF; however, even if a novel tubular injury biomarker is 100% sensitive and 100% specific, it may appear inaccurate when using serum creatinine as the gold standard. Thus we still have to be aware of the potential limitations as well as we have to bear in mind that the apparent diagnostic performance of a biomarker depends not only on its ability to detect injury, but also on disease prevalence and the sensitivity and specificity of the imperfect gold standard^25^.

**Figure 1:**
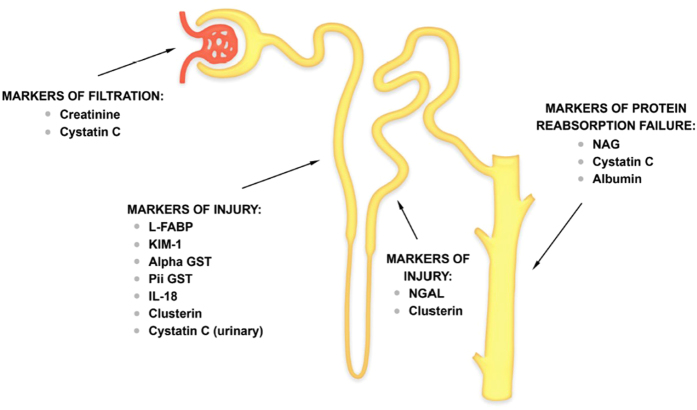
Novel biomarkers indicative of acute kidney injury.

Typically, two broad classes of renal biomarkers of renal damage are distinguish – markers of kidney function (represented by cystatin C) and markers of kidney injury (for example urine and serum neutrophil gelatinase-associated lipocalin [NGAL], kidney injury molecule 1 [KIM-1], liver-type fatty-acid binding protein [L-FABP], interleukin-18 [IL-18]^27^(Fig. 1).

### Neutrophil gelatinase-associated lipocalin (NGAL)

NGAL (also known as siderocalin or lipocalin-2) discovery is the result of “fishing expedition” (cDNA microarray) and NGAL turned out to be the most upregulating gene in kidney (“the biggest fish”)^28^. NGAL is a protein composed of 178 amino acids with the molecular weight of 25 kDa, isolated for the first time in the 90 s last century of human neutrophils as a molecule associated with gelatinase^29^. All NGAL’s functions and mechanisms are not completely elucidated. NGAL is secreted in kidney’s tubular cells and hepatocytes as well as in immunological, gastrointestinal and pulmonary tract cells^29^. NGAL belongs to the group of acute phase proteins. Moreover it is involved in apoptosis^30^. After nephrotoxic or ischemic injury NGAL is notably accumulated in kidney cortical tubules, blood and urine^31^. NGAL is excreted in the urine through the epithelial cells of ascending limb of loop of Henle and connecting tubules. It is considered that NGAL is involved in chelation of iron complex thereby inhibiting bacterial growth^32^.

By references to numerous research the usefulness of NGAL in an early diagnosis of AKI has been proven in common clinical situations such as cardiac surgery-associated AKI^33^,^34^,^35^, contrast-induced AKI^36^,^37^,^38^, AKI in critical care^39^,^40^. Mishra *et al.* have shown that NGAL staining intensity significantly correlates with the development of DGF^41^. Parikh *et al.*^42^ demonstrated an earlier prediction of DGF using the measurement of urine NGAL in comparison to other clinical parameters and definition that are currently in use. It was also confirmed by Hall *et al.*^43^ studies, which showed that urinary NGAL is an early, noninvasive and accurate biomarker of the necessity for dialysis during the first week after renal transplantation. Moreover there is a correlation between plasma NGAL level and DGF succeeding kidney transplantation from donors after cardiac death^44^,^45^. In our studies we found that before transplantation, serum NGAL was related to creatinine and cystatin C^46^. At each time point, serum NGAL was related positively to ser um creatinine, cystatin C, and negatively to urine volume. In adidtion, in patients with delayed graft function, there was no fall in serum NGAL or cystatin C. Therefore, we concluded that NGAL should be investigated as a potential early, sensitive marker of kidney impairment/injury, which might provide an additional accurate measure of kidney impairment in CKD and among transplant recipients, particularly at advanced stages. In the recent studies, Buemi *et al.*^47^ studied the predictive value of urinary and plasma NGAL in both living and deceased kidney transplant recipients. Recipient plasma NGAL performed better than urinary NGAL in terms of predicting DGF occurrence, whereas donor plasma NGAL and urinary NGAL values did not influence the time needed to reach serum creatinine levels of <2 mg/dl after transplantation. In addition, recipient plasma NGAL values obtained 24 and 48 h after transplantation, but not urinary NGAL values, were found to be a significant predictor of graft function recovery.

At the beginning, NGAL was identified by Western Blot technique. In many studies, NGAL started to rise at 2 hours after the injury, peaking at 8–12 hours and returned to normal after 24–48 hours^33^,^34^,^35^,^36^,^37^,^41^,^42^,^43^, however, data on the time course of NGAL changes in kidney allograft recipients are limited. Nowadays in widespread use are ELISA assays such as BIOPORTO. The clinical measurement of plasma NGAL is available on Triage^®^ NGAL device (Inverness, Inc, San Diego, CA, USA). The advantage of this kit is to obtain quantitative results in 15 min and requiring a microliter sample^48^. Furthermore the clinical platform Architect^®^ analyzer (Abbott Diagnostics, Abbott Park, IL, USA) is able to perform an easy and non-invasive test - urinary NGAL immunoassay with the results available in 35 minutes^49^.

### Kidney injury molecule 1 (KIM-1)

Kim-1 (also known as Tim-1 – T-cell immunoglobulin and mucin-containing molecule), firstly described by Ichimura *et al.*^50^ in 1998, is a type 1 transmembrane protein with two extracellular domains: 6-cysteine immunoglobulin-like and mucin-like O-glycosylated protein. It has also cytoplasmic and transmembrane domains. KIM-1 is hardly detected in normal kidney tissue and urine^51^. However, it is expressed at very high levels in proximal tubules shortly after the kidney injury such including acute kidney injury caused by ischemia, nephrotoxic drugs, as well as chronic kidney disease and acute/chronic renal transplant dysfunction^51^.

Increase in KIM-1 is observed particularly in the S3 segment of proximal tubule. There is no evidence of KIM-1 expression in the glomerulus, peritubular interstitial cells and inner medullary cells^50^,^52^. The quantitative marker of KIM-1 measured in urine by immunoassay is the extracellular ectodomain of molecular weight 80–90 kDa^52^. It is cleaved by metalloproteinase from the surface of damaged activated tubular cells.

KIM-1 has a numerous features of ideal biomarker such as absence in normal kidneys and increasing expression after renal injury, significant relationship between urine and tissue level of KIM-1 and correlation with the severity of renal dysfunction^51^. KIM-1 may also help distinguish acute tubular necrosis from other causes of acute kidney injury, both by measuring the urine and expression in renal tissue^53^,^54^. However, according to Marcus *et al.*^55^ there was no statistically significant difference between urinary KIM-1 level in patients with delayed and normal graft function. In both groups it remained in high level. Zhang *et al.*^54^ demonstrated that expression of KIM-1 preceded the appearance of morphological changes in tubules and positively correlated with degree of kidney damage. They also indicated the possibility of dual role of KIM-1 – beyond tubular injury biomarker it is involved in regeneration process and as such may be a potential marker of renal repair.

KIM-1 peaked at 2–3 hours in patients undergoing cardiac surgery^56^,^57^, returning to baseline within 24–48 hours, but data in kidney transplant recipients in this regards are inconclusive^43^.

A rapid direct immunochromatographic lateral flow 15-min assay for detection of urinary Kim-1 (rat) or KIM-1 (human) has been developed^58^. Using this assay, more urinary KIM-1 was detected in the urine of patients with AKI than in the urine from healthy volunteers. Recently, Filed *et al.*^59^ studied urine samples from 182 brainstem dead multi-organ donors (all of whom donated hearts that were transplanted) were analyzed for a Luminex(™) panel of biomarkers linked with AKI, including KIM-1, NGAL and others. They found that donor urinary KIM-1 levels were significantly higher in donors whose kidneys displayed aberrant early function (p = 0.011). In addition, they also stated that the availability of a lateral flow device (Renastick(™) ) for KIM-1 that also demonstrated higher urinary KIM-1 levels in donors whose kidneys show aberrant initial function (p = 0.03), made KIM-1 a potential indicator of AKI that may merit further evaluation for its application at the donor bedside. Biomarker dipstick, such as for KIM-1, may thus provide a sensitive and accurate detection of kidney injury in clinical trials and then in clinical practice. However, in case of DGF urinary KIM-1 appears to be useless.

### Interleukin-18 (IL-18)^60^

Interleukin-18 (IL-18) is a proinflammatory cytokine with a molecular weight of 18kDa^60^. It is a part of the IL-1 family of cytokines and is produced in an inactive form in which it remains until caspase-1 activated by ischemia cleaves it into its active form. IL-18 is especially abundant on macrophages but also on dendritic cells, monocytes and kidney epithelial cells. In healthy individuals – serum and urinary levels of IL-18 generally remain low and significantly increased in states such as renal tissue damage^61^.

It participates in different diseases such as ischemia-reperfusion injury, minimal change nephrotic syndrome or transplant rejection, but above all IL-18 is a mediator and a biomarker of ischemic tissue damage causing AKI. Furthermore it is seems to be specific to this particular type of kidney injury which was established by Parikh *et al.*^62^ in the study showing increased IL-18 levels in patients treated for AKI but not in those with urinary tract infections, prerenal azotemia, chronic kidney diseases or healthy controls. A different study suggests that knockout mice deficient in caspase enzyme, tend to develop less severe tubular necrosis than their wild-type cousins^60^. It has been demonstrated that in patients with minimal change nephrotic syndrome urinary IL-18 levels remained in correlation with proteinuria and the severity of the disease itself^63^.

Another fact in favor of IL-18 as a future biomarker is that relatively affordable, accessible and fast commercial ELISA kits to measure urine IL-18 levels are already available by Raybiotech Co Ltd. in more than 50 countries.

Alike urine NGAL levels, IL-18 urine levels were significantly increased especially in the first 24 hours (peaking at 6 hours), following cardiac surgeries and successfully predicted AKI and its progression^64^.

However several studies have demonstrated a close association between systemic inflammation and urinary IL-18 levels^65^. Patients treated in intensive care units had higher urinary IL-18 concentration levels than healthy controls^66^,^67^ which may impact its predictive role as a biomarker.

### Liver-type fatty-acid binding protein [L-FABP]

Fatty-acid protein bindings (FABPs) are a family of nine, organ specific cytoplasmatic proteins that are involved in the intracellular lipids transport. There are specific types in liver, intestinal, heart muscle, adipocyte, epidermal, ileal brain, myelin and testis. Their exact role and biological mechanism of action remains barely understood^68^. L-FABP is synthesized mostly in the liver and due to its small molecular size (14 kDa) it can be filtered through glomerulus and reabsorbed by epithelial cells in proximal tubule partly through megalin-dependent system. L-FABP is not detected in the urine of healthy individuals. However, in animal models, during kidney injury caused by ischemia the reabsorption of L-FABP decrease and it occurs in urine^69^,^70^. It was also confirmed in several human studies. In the study on adults undergoing CBP urinary L-FABP increased significantly after 4 hours following surgery, whereas serum L-FABP started to increase after 12 hours postoperatively^71^, indicating that urinary L-FABP was mostly determined by proximal tubule injury. Doi *et al.*^72^ reported that in patient with septic shock complicated with AKI higher concentration of urinary L-FABP at the time of admission correlated with greater mortality. Recently, Yang *et al.*^73^ assessed urinary NGAL and L-FABP levels at 0 hours, 2 days, and 6 days after kidney transplantation (KT) and followed patients for up to 2-years after kidney transplantation. They found that high 0-hour L-FABP (P = 015) and acute rejection (P = .006) were independent factors predicting poor long-term graft function. They also suggested that urinary L-FABP might be a useful predictor of adverse long-term outcomes in kidney transplant recipients. Additionally, L-FABP is simply measured by enzyme-linked immunosorbent assay (ELISA)^74^ that are commercially available.

### YKL-40

YKL-40 is a 40 kDa heparin- and chitin-binding glycoprotein^75^. The abbreviation YKL-40 is based on the one letter code for the first three N-terminal amino acids, tyrosine (Y), lysine (K) and leucine (L) and the apparent molecular weight of YKL-40^76^. Recently, urinary and serum YKL-40 levels are reported to be elevated in patients with DGF^77^. Schmidt *et al.*^77^ measured urinary and blood YKL-40 values at different time points by level of allograft recovery. They found that patients with delayed graft function has highest serum YKL-40 when compared with patients with slow or immediate graft function 2 days after transplantation. They also studied urinary YKL-40 and observed that it was markedly elevated in patients with delayed graft function. It may be due to the fact that YKL-40 was secreted by neutrophils and monocyte/macrophages as a part of the innate immune response to injury.

They suggested that YKL-40 could be as a possible biomarker to identify patients at greatest risk of sustained renal failure following transplantation.

### Clusterin

Very recently, Pianta *et al.*^78^ studied the utility of clusterin for prediction of DGF (hemodialysis within 7 days of transplantation) was compared with urinary interleukin (IL)-18, neutrophil gelatinase-associated lipocalin (NGAL), kidney injury molecule-1, serum creatinine, and clinical variables. Both NGAL and clusterin are synthesized in the proximal as well as distal tubules^79^. They found that at 4  r after reperfusion, anuria was highly specific, but of low sensitivity for detection of DGF. At 4 hr, receiver operating characteristic analysis suggested that urinary clusterin, IL-18, kidney injury molecule-1, and NGAL concentration were predictive of DGF. Moreover, after adjusting for preoperative clinical variables and anuria, clusterin and IL-18 independently enhanced the clinical model for prediction of DGF. Finally they conclude, that both urinary clusterin and IL-18 are useful biomarkers and may allow triaging of patients with DGF within 4 hr of transplantation.

### Cystatin C

**Figure 2:**
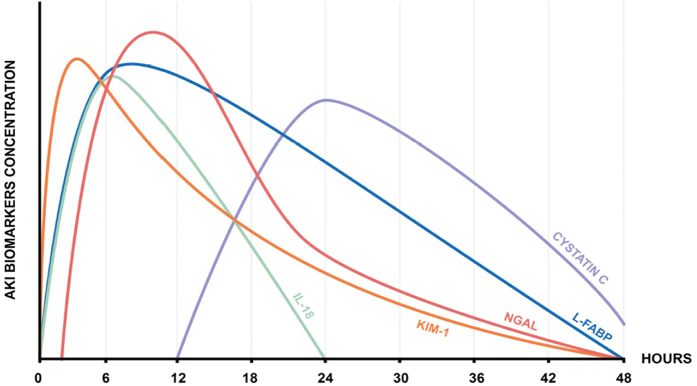
Time course of novel urinary biomarkers.

So far we discussed markers of kidney injury, while cystatin C represents markers of kidney function. It is is a polypeptide chain with 120 amino acid residues of a basic low molecular mass protein (13,359) freely filtered by the glomerulus and subsequently reabsorbed by the proximal tubule where it is catabolized^80^. As it is not secreted by the renal tubules as creatinine^81^, some limitation of creatinine (effect of muscle mass, diet, sex, tubular secretion) are not taken into consideration. However, cystatin C is affected by hyper- and hypothyroidism, and inflammation^82^,^83^. It was not find to be superior to creatinine in estimating kidney function^84^. Herget-Rosenthal *et al.*^85^ reported that serum cystatin C is a useful detection marker of acute kidney injury and might detect it one or two days earlier than creatinine. It usually increases after 12 hours, peaking at 24 hours, then returns to baseline^86^,^87^. Urinary cystatin C, which represents tubular injury was found to be superior to conventional and novel plasma markers in the early diagnosis of acute kidney injury following adult cardiac surgery^88^. In our study we found that serum NGAL decreased significantly as early as 1 days after kidney transplantation, prior a fall in cystatin C and in creatinine^46^. At each time point serum NGAL was related positively to serum creatinine, cystatin C, and negatively to urine volume. In 4 patients we observed a delayed graft function-DGF, in all of these patients we did not observe a fall in serum NGAL, creatinine and cystatin C. At any time serum NGAL, cystatin C and creatinine were significantly higher in patients with DGF when compared to patients without DGF. However, urinary NGAL has a limited usefulness in anuric patients. In the Fig. 2 time course of urinary biomarkers is presented based on the available literature.

### Summary

The complexity of the pathophysiology of acute tubular necrosis/acute kidney injury makes that there is a low probability of finding one sensitive and specific biomarker, rather the entire panel, with NGAL being the most studied^89^. All of the listed putative biomarkers have their strengths and weaknesses. Biomarkers connections are likely to be needed, but they are not well characterized. A limitation of the prevalence of their use is high cost of production and validation, as well as lack of standardized laboratory platforms to assess them. Moreover, current studies are based on relatively small numbers of patient and events. Besides, most research was carried out using frozen samples, thus affecting their quality. Another limitation is the absence of pharmacoeconomic analysis.

However, all these biomarkers reflect kidney injury, and search for biomarkers able to predict acute kidney injury and delayed graft function continues.

## Additional Information

**How to cite this article**: Malyszko, J. *et al.* Biomarkers of delayed graft function as a form of acute kidney injury in kidney transplantation. *Sci. Rep.* **5**, 11684; doi: 10.1038/srep11684 (2015).

## Figures and Tables

**Table 1:** Potential usefulness of biomarkers in kidney transplantation.

marker	abbreviation	Sample		Usefulness in kidney transplantation
		serum	urine	
Neutrophil gelatinase-associated lipocalin^41^,^42^,^43^,^44^,^45^,^46^,^47^	NGAL	+	+	+
Kidney injury molecule 1^59^	KIM-1	−	+	+/−
Interleukin-18^42^,^43^,^78^	IL-18	+	+	+
Liver-type fatty-acid binding protein^73^	L-FABP	−	+	+
40 kDa heparin- and chitin-binding glycoprotein^74^	YKL-40	+	+	+
Cystatin C^46^	Cys C	+	+	+
Clusterin^78^	clusterin	+	+	+
